# Oxygen Effect on 0–30 eV Electron Damage to DNA Under Different Hydration Levels: Base and Clustered Lesions, Strand Breaks and Crosslinks

**DOI:** 10.3390/molecules29246033

**Published:** 2024-12-21

**Authors:** Yingxia Gao, Xuran Wang, Pierre Cloutier, Yi Zheng, Léon Sanche

**Affiliations:** 1 State Key Laboratory of Photocatalysis on Energy and Environment, Fuzhou University, Fuzhou 350116, China; gyxmyr3719@163.com (Y.G.); xr_wang1215@163.com (X.W.); yi.zheng@usherbrooke.ca (Y.Z.); 2 Department of Nuclear Medicine and Radiobiology and Clinical Research Center, Faculty of Medicine and Health Sciences, Université de Sherbrooke, Sherbrooke, QC J1H 5N4, Canada; pierre.cloutier@usherbrooke.ca

**Keywords:** oxygen effect, low-energy electrons, DNA damage, ionizing radiation, humidity

## Abstract

Studies on radiosensitization of biological damage by O_2_ began about a century ago and it remains one of the most significant subjects in radiobiology. It has been related to increased production of oxygen radicals and other reactive metabolites, but only recently to the action of the numerous low-energy electrons (LEEs: 0–30 eV) produced by ionizing radiation. We provide the first complete set of G-values (yields of specific products per energy deposited) for all conformational damages induced to plasmid DNA by LEEs (G_LEE_ (O_2_)) and 1.5 keV X-rays (G_X_(O_2_)) under oxygen at atmospheric pressure. The experiments are performed in a chamber, under humidity levels ranging from 2.5 to 33 water molecules/base. Photoelectrons from 0 to 30 eV are produced by X-rays incident on a tantalum substrate covered with DNA. Damage yields are measured by electrophoresis as a function of X-ray fluence. The oxygen enhancement ratio G_LEE_(O_2_)/G_LEE_(N_2_), which lies around 2 for potentially lethal cluster lesions, is similar to that found with cells. The average ratio, G_LEE_(O_2_)/G_X_(O_2_), of 12 for cluster lesions and crosslinks strongly suggest that DNA damages that harm cells are much more likely to be created by LEEs than any other initial species generated by X-rays in the presence of O_2_.

## 1. Introduction

Secondary electrons (SEs) are the most abundant species produced by high-energy radiation (HER) [1,2,3,4]. In various materials, including living organisms, most of these electrons are created with energies below 30 eV; they are usually referred to as low-energy electrons (LEEs) [3,5,6]. Thus, at the very beginning of the energy deposition process, the two most numerous reactive species are positive ions and LEEs. During the last two decades, a considerable number of experiments and theoretical calculations have demonstrated that LEEs could cause considerable damage to DNA [5,6,7,8,9]. For about the same period, experimental and theoretical attempts have been made to determine the mechanisms causing specific damages to this molecule and its components, ranging from simple subunits such as DNA bases, to relatively long oligonucleotides [9,10,11,12,13,14], and up to eukaryotic-cell nuclear DNA [15]. It is now established that below 15 eV, LEEs localize temporarily on the DNA subunits to form transient anions (TAs) [10,12,16]. These TAs can damage DNA mostly via dissociative electron attachment (DEA), i.e., by dissociating into a stable anion and one or more radical fragments. Alternatively, a TA can autoionize and cause a bond rupture by leaving a DNA component in a dissociative electronically excited state [17]. LEEs can also transfer from one DNA subunit to another, particularly from a base to the phosphate group, where they can cleave the C–O bond and break a strand [9,16,18]. If DEA occurs from attachment of an autoionizing electron that left a site of bond cleavage, then two very close (clustered) lesions can occur with a single LEE [16,19].

At the experimental level, the identification and mechanisms of action of TAs in DNA and its components have been mainly deduced from damage yield functions and electron energy-loss spectra, i.e., the observation of strong peaks in the electron energy dependence of damage yields and energy losses was assigned to the formation of TAs [7,16,20]. However, such features can only be measured under vacuum, since they require exposure of the target to monoenergetic LEEs. This condition limits investigations of DNA damage to the direct effect of LEEs. In other words, from the vacuum experiments, we gain considerable knowledge on electron–DNA interactions, but we do not have any information on the influence of the cellular environment on the mechanisms involved. The environment of the cell is complex. The various molecules are adjacent to DNA, including histones, proteins, salts, oxygen, and water. The latter is the most abundant molecule, and it is implicated in cellular construction and functionalization [21,22]. Water radiolysis produces cations, anions, hydroxyl radicals, solvated electrons, and hydrogen radicals that can further react with DNA [21]. These species emanate from the interaction of the initial positive ions and SEs with water. However, in cells, the indirect effect of reactive species on DNA arises not only from water, but also from the interaction of HER with any other surrounding biomolecules [21]. More generally, the indirect effect of HER, which constitutes an essential counterpart of the complete radiobiological mechanism of DNA damage [23], can be considered as any contribution arising from a combination of the environment with the radiation. For example, organic ions [24,25], amino acids [26,27,28,29], and proteins [30,31,32] could protect DNA against HER and LEE-induced damage.

Experimental [33,34,35,36] and theoretical [37,38,39,40] evidence have contributed to understanding LEE-induced damage in the presence of water, namely that water around DNA modifies the TA manifold, the corresponding decay channels [41], and electron diffraction [42,43]. Such modifications have been observed in electron attachment to clusters containing DNA constituents or their analogs [44,45,46] and with plasmid DNA deposited on a metal substrate emitting low-energy photoelectrons (LEPEs) produced by X-rays incident on the metal [47,48,49]. The later experiments were performed in N_2_, O_2_, and N_2_O gases at standard atmospheric temperature and pressure (SATP) under dry and hydrated conditions [50,51,52,53,54,55]. The results from these investigations have been summarized in a recent article on the indirect effect of LEEs on plasmid DNA under various hydration levels of nitrogen gas at SATP [56].

In the initial definition, the radiation damage caused by the HER interaction with DNA corresponded to the damage induced directly in the molecule, plus that indirectly arising from the reactive species generated by HER’s interaction with surrounding water molecules [37,57]. At the time, LEEs were not considered to damage DNA [57]. Presently, the damage caused to DNA by LEEs becomes part of the direct effect [58,59], whereas DNA lesions arising from reactive species produced by LEEs are assigned to the indirect effect [7,23,51], i.e., it does not depend on where LEEs are created. In investigations related to the direct and indirect effect of radiation, yields are usually expressed as G-values, which correspond to the number of a given lesion or total damage per energy deposited in the target.

Studies on radiosensitization of cellular damage by O_2_ began about a century ago. Since then, O_2_ radiosensitization has remained one of the most significant subjects in radiobiology [60,61,62]. Exposure of experimental animals and cells to large concentrations of O_2_ increases radiation damage, e.g., in mammalian cells an average oxygen enhancement ratio (OER) of about 2 is observed, as computed from Table S1 in the Supplementary Materials. The toxic effects of cell exposure to O_2_ were interpreted as due to the reaction of O_2_ with damage sites within DNA, a phenomenon called “Oxygen fixation” [62,63,64]. The toxicity was related to an increased production of oxygen radicals or other reactive metabolites derived from DNA and oxygen [63,65]. More precisely, short-lived free radicals (DNA•) arising from the HER interaction with DNA react with nearby O_2_ to generate peroxyl radicals (DNA-OO•) [66,67]. This chemical modification of DNA is more difficult for the cell to repair [67]. As one of the main and most toxic reactive oxygen species, excessive superoxide radicals ($O_{2}^{•-}$)in the body react with proteins, DNA, and lipids, irreversibly damaging cellular components and disrupting cellular metabolism function [63,64,65,68,69]. Meanwhile, $O_{2}^{•-}$ can be transformed into the highly toxic OH^•^ radical through disproportionation reactions via intracellular superoxide dismutase and subsequent reactions, thereby dramatically aggravating oxidative damage and improving anticancer efficacy [65,70,71]. The results of Li et al. show that $O_{2}^{•-}$ and OH^•^ radicals synergistically disrupt the integrities of cellular lysosomes and nuclei, subsequently triggering cancer cell apoptosis to enhance therapeutic efficiency [72].

Although the oxygen effect on irradiated cells and DNA has been known for about a century, it is only recently that the considerable contribution to this effect by the most numerous SEs produced by HER (i.e., LEEs) has been investigated by Alizadeh et al. [52,54,55] and Mirsaleh-Kohan et al. [73]. Recently, measurements of LEE-induced damages to DNA have been extended to lesions other than prompt inter-duplex crosslinks (CLs), single strand breaks (SSBs), and double strand breaks (DSBs) to include base damages (BDs), BD-related inter-duplex CLs (BD-CLs), and non-DSB clustered damages (NDCDs) [56,74]. Advances have also been made in the instrumentation and thin DNA film deposition [75]. These have improved the accuracy and sensitivity of all detectable lesions by electrophoresis, allowing further treatment with enzymes of the irradiated samples that reveals BD-related lesions [56]. Considering these advances, we can provide a complete set of G-values for all types of damages to plasmid DNA measurable by electrophoresis. Under identical conditions, we measure the DNA lesions induced by 1.5 keV photons and the LEPEs they produced from a tantalum (Ta) substrate in an O_2_ atmosphere at SATP. G-values are reported for Γ = 2.5, 10, 20, and 33, where Γ is the number of water molecules per nucleotide. Γ = 2.5 corresponds to dry DNA, whereas Γ of 10, 20, and 33 correspond to the first (2.5 < Γ < 10) and second (10 < Γ ≤ 20) hydration layer and DNA surrounded by bulk-like water, respectively, i.e., atmospheric relative humidities of 0%, 50%, 80%, and 100% [76]. The LEPE energy distribution from the Ta substrate resembles that created by the sum of all SEs created by HER [4,56]. Our results are compared to previous ones on SSBs and DSBs obtained in similar experiments with plasmid DNA in air and pure O_2_ at Γ = 2.5 and 33 [52,54], as well as those obtained from DNA films irradiated with HER.

## 2. Results and Discussion

### 2.1. Yields of DNA Damages Induced by X-Rays and LEPEs in an Oxygen Atmosphere Under Different Hydration Levels

**Figure 1:**
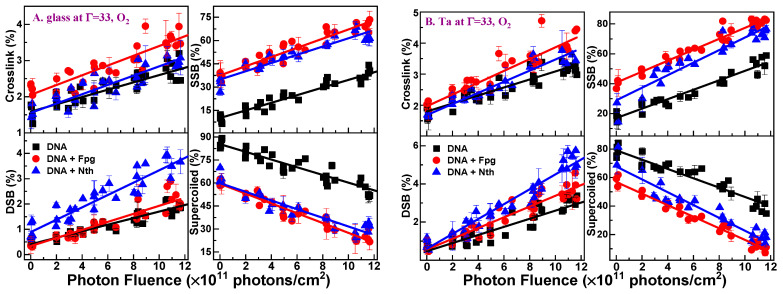
Exposure–response curves for crosslinks, SSBs, DSBs and loss of supercoiled in 5-monolayer DNA films (■) induced by X-rays on glass (**A**) and Ta (**B**) under 100% humidity (Γ = 33), together with the enzymatic treatments with Fpg (●) and Nth (▲). Exposure–response curves were fitted with a linear function. Each data point is the result of three identical bombardment procedures, and the error bars are the standard deviation.

Representative exposure–response curves for different DNA conformations resulting from X-ray irradiation of plasmid DNA surrounded by O_2_ at SATP and 100% relative humidity (Γ = 33) are shown in Figure 1. The curves recorded on glass and Ta appear in A and B, respectively. The black squares denote the yields of the specific conformations measured by electrophoresis without enzyme treatment, while the blue triangles and red circles correspond to the yields after treatment of the samples with Nth (▲) and Fpg (●) enzymes, respectively. Similar curves were also recorded at Γ = 2.5, 10, and 20. All exposure–response curves indicate that, within experimental errors, the yields per photon·molecule·cm^−2^ of respective lesions are linear with X-ray fluence, i.e., they are induced by a single event. The data of Figure 1 consist of 961 points from four irradiation experiments, with an average of 30 samples for one exposure at Γ = 33. For all hydration levels, the total number of measurements amounts to 9048 (12,064 including heat liable). The data points at zero fluence represent the initial plasmid conformations, plus the yields of lesions created by manipulations. These lesions do not influence the determination of the G-values, which arise from the slope of the fluence–response curves.

**Figure 2:**
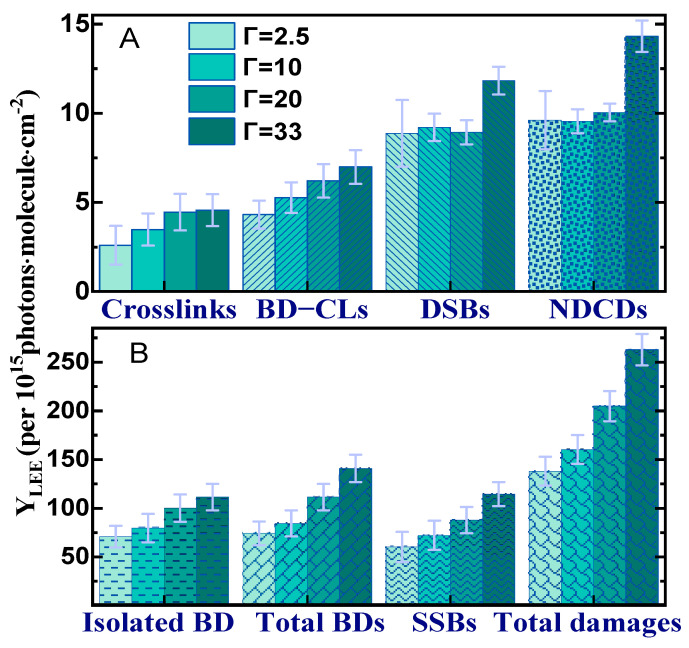
The yields (per 10^15^ photons·molecule·cm^−2^) of (**A**) CLs, BD−related crosslinks (BD−CLs), DSBs, and non-DSB cluster damages (NDCDs) and (**B**) isolated BDs, total BDs, SSBs, and total DNA damages induced by LEPEs for different hydration levels Γ under an O_2_ atmosphere at SATP. Γ is the number of water molecules per nucleotide. The error bars are the standard deviations from six identical measurements.

Figure 2 shows as histograms the yields of the lesions induced by the LEE interaction with DNA (Y_LEE_) in an oxygen atmosphere under the four hydration levels. The yields in Figure 2B are at least one order of magnitude larger than those shown in Figure 2A. Apart from DSBs and NDCDs, whose yields become larger only at Γ = 33, Y_LEE_ increases progressively as a function of hydration. As expected, the increase in single damages is reflected in the total yields, indicating that in the presence of oxygen, the most prominent DNA damages are proportional to the magnitude of Γ. A similar behavior was found in an N_2_ atmosphere, but only up to Γ = 20 [56].

### 2.2. G-Values for DNA Damage Induced by X-Rays and LEPEs in an Oxygen Atmosphere Under Different Hydration Levels

**Figure 3:**
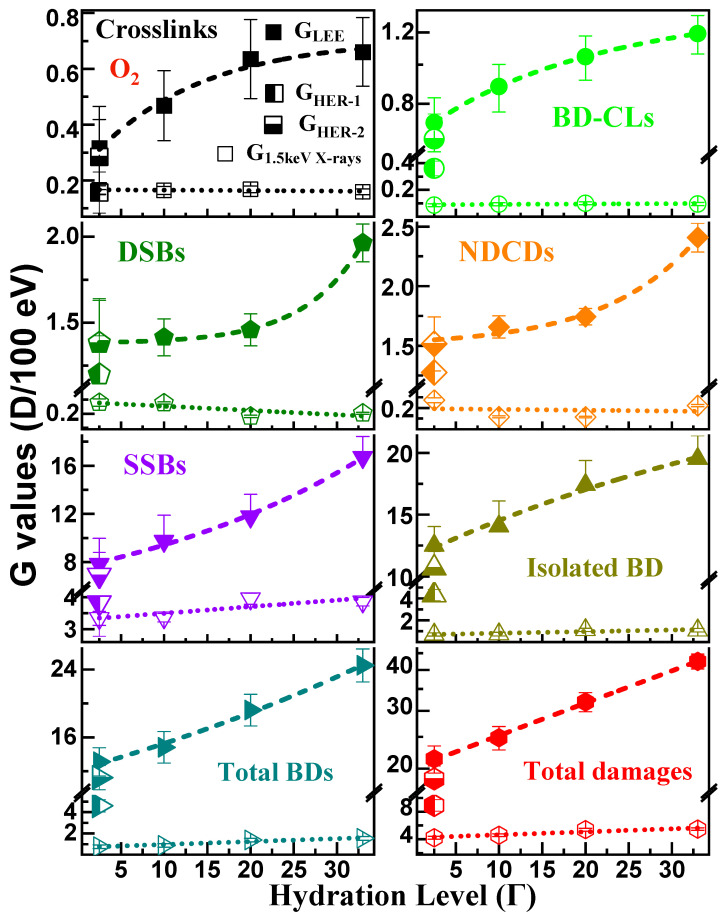
G-values (number of a specific damage D per 100 eV of energy deposited) recorded under O_2_ at SATP, as a function of hydration level Γ, for LEPEs (G_LEE_, full symbols) and 1.5 keV X-rays (G_X_, open symbols). The G_HER-1_ and G_HER-2_ values were obtained at Γ = 2.5 for electron distributions created by the initial and all secondary electrons produced by HER, respectively (half-filled symbols). The G-values are provided for crosslinks (CLs), SSBs, DSBs, BD-related CLs, isolated BDs, non-DSB cluster damages (NDCDs), total BDs, and total DNA damages. The dashed lines are splined curves fitted to the data points. The error bars are the standard deviations resulting from six identical experiments.

The G-values obtained from the yields of Figure 2 are listed in Table 1 and displayed graphically in Figure 3. The raw data before applying the correction factors CF are provided in Table S2. All results produced so far on thin films irradiated with different radiation sources are listed in Table S3. For all types of DNA damages, the G_LEE_ of Figure 3 are seen to increase as a function of hydration levels, while G_X_ does not exhibit considerable changes, except for a marked increase in SSBs, and a surprising decrease of cluster lesions (i.e., DSBs and NDCDs) with increasing Γ. G_LEE_ for CLs and BD-CLs exhibit an exponentially saturating behavior, as previously observed in an N_2_ atmosphere for all lesions [56]. Here, the opposite behavior is observed for DSBs and NDCDs, i.e., their G-values rise exponentially with increasing Γ. As shown by the enhancement ratio of G_LEE_-values in going from Γ = 2.5 to 33, these cluster damages are the least affected by the presence of water. Isolated BDs and total BDs have a nearly linear growth.

**Figure 4:**
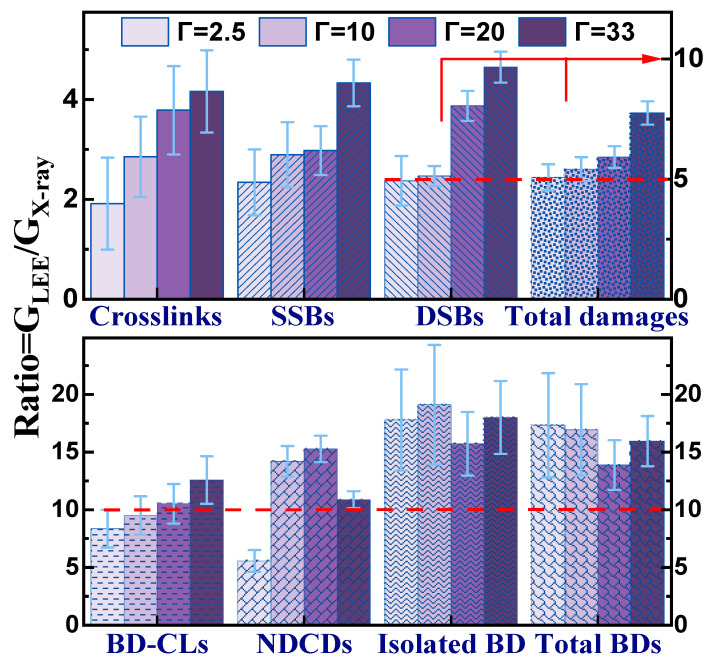
Dependence of the ratio G_LEE_ to G_X_ on the level of hydration for all measured damages in dry and humid DNA films under O_2_ at SATP. The red arrow indicates the right scale for DSBs and the sum of all measured damages. The error bars are the standard deviations obtained from the division of the two G-values.

The contrasting behavior of G_LEE_ with increasing hydration compared to that of G_X_ and the large differences in their magnitude are more obvious in Figure 4, which displays ratios (G_LEE_/G_X_) ranging from 2 to 19. At 100% humidity, G_LEE_ values are at least four times higher than those of G_X_. Remarkably, the ratio G_LEE_/G_X_ doubles with added water for cluster lesions and reaches maxima above 14 for total and isolated BDs, BD-CLs, and NDCDs. Obviously, LEEs are much more effective in damaging DNA than 1.5 keV X-rays for the same energy deposited. *Moreover, the maximum ratios (G_LEE_/G_X_) of 10, 15, and 13 for DSBs, NDCDs, and BD-CLs, respectively, strongly suggest that potentially lethal lesions are much more likely to be created by LEEs than by X-rays in the presence of O_2_.* Apart from the ratios of BDs that do not depend on Γ, the increasing effectiveness of LEEs to induce DNA damage with increasing humidity indicates that these electrons could play a significant role in the production of the abundant free radicals induced by HER in oxygenated water. One of the abundant reactive species from DEA to H_2_O, OH^•^, could interact with DNA radical (DNA^•^) also created via DEA, leading to an oxidized lesion, e.g., OH^•^ could react with a C3′-radical from LEE-damaged DNA, yielding the 3′-hydroxyribose adduct. Hence, the action of these radicals could synergistically combine with the direct impact of LEEs on DNA to produce the detected damages.

Obviously, X-rays and LEPEs do not interact with DNA in the same manner. This is not surprising, since LEPEs induce DNA damage principally via the formation of TAs, whereas X-rays mainly ionize DNA and its surrounding, initially producing a cation and a wider energy spectrum of SEs containing many non-resonant electrons. Thus, compared to the action of cations, TA formation considerably boosts the formation of damages in the presence of oxygen, particularly DEA leading to cluster lesion and CLs. Considering the linearity of the exposure–response curves, this result corroborates experimental [16] and theoretical [77] studies, suggesting that a single LEE can create a cluster lesion in DNA via the formation of core-excited resonances, i.e., TAs composed of two excited electrons quasi-bound to a positive ion core.

In the range 0–30 eV, the energy distribution of electrons striking a target influences the magnitude of G-values [56]. However, once the G-values for a given distribution (e.g., Z(E), Figure S1) are known, those for distributions from other sources can be mathematically generated, as explained in the Supplementary Materials. This mathematical transformation requires the electron energy dependence of the damage yields in the target to be known [56]. Presently, such functions are known for dry plasmid films [16]. Hence, we can provide in Table 1 the G-values G_HER-1_ and G_HER-2_ of dry DNA in an oxygen atmosphere calculated for two typical LEE energy distributions in water produced by HER. These two distributions Z_HER-1_ and Z_HER-2_ are provided in Figure S1. Z_HER-1_ corresponds to the initial SE energy distribution created at the instant of the first interaction of a fast charged particle in water [3], and Z_HER-2_ corresponds to the energy spectrum of the total number of LEEs produced by a fast charged particle in water within approximatively one femtosecond after the initial interaction [4]. As seen from the comparison of the G-values in Table 1, the measured G-values at Γ = 2.5 lie close to those calculated with the latter energy distribution and thus represent fairly well the yields of specific damages per energy deposited by all SEs produced by HER.

**Figure 5:**
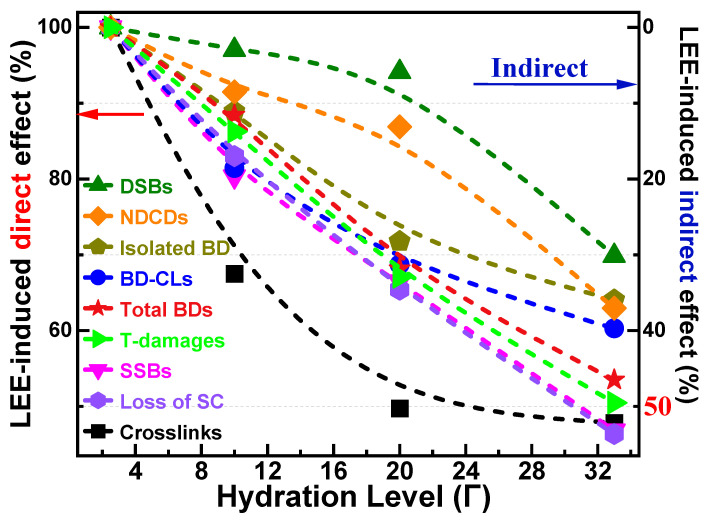
Percentage (%) of the direct and indirect effects contributing to G_LEE_ (D/100 eV) for CLs, SSBs, DSBs, BD-related CLs, isolated BDs, NDCDs, total BDs, and total (T) DNA damages induced by LEEs as a function of hydration level under an O_2_ atmosphere at SATP. The left scale corresponds to the direct effect, whereas the right one (blue arrow), which is inverted, denotes the indirect contribution.

G-values can also be represented as a proportion of the direct and indirect effect of the action of radiation, including that of LEEs. For the latter, these can be defined as contributions to the damage arising from the direct LEE interaction with DNA, plus those from the reaction with DNA of the reactive species produced from the LEE interaction in water both with and without the participation of oxygen. It should also be kept in mind that the direct effect can be modulated by the DNA conformational modifications arising from hydration. Within the approximation that the indirect effect of LEEs corresponds to damage linearly added to the direct effect, we can calculate percentage of the direct effect for each value of Γ on DNA damages from dividing G_LEE_ (direct Γ = 2.5) by the respective G_LEE_ (Γ = 10, 20, 33), as shown in Figure 5. Interestingly, hydration influences differently the contribution of indirect effect on specific lesions. For example, at complete hydration, the indirect effect is slightly larger than 50% in the case of prompt CL formation, whereas for DSBs it is only 30%. The other lesions have contributions between these extremes.

Furthermore, the dependence on Γ of the direct or indirect effects shows a different behavior depending on the lesion (Figure 5), i.e., CLs exhibit an exponentially saturating behavior of indirect damage, whereas an opposite trend is observed for DSBs and NDCLs, with a strong increase from Γ = 20 to 33. SSBs, the sum of BDs, and the total of all lesions display a nearly linear dependence on hydration. The direct effect contributes to 86%, 67%, and 50% of the total damages at Γ = 10, 20, and 33, respectively. The present behaviors of DSBs, NDCDs, SSBs, and BDs were not observed in an N_2_ atmosphere, where the magnitude of the indirect effect quasi-exponentially saturates vs. Γ for all lesions [56].

### 2.3. The Oxygen Enhancement Ratio (OER): Effect of O_2_ vs. N_2_ on G-Values

In cells, the oxygen effect is measured as the enhancement of radiotoxicity from exposure to O_2_ as compared to anaerobic conditions [69,78]. In our simpler conditions, we reference the present G-values recorded in O_2_ to those previously measured in a pure nitrogen atmosphere to derive the oxygen enhancement ratio (OER). According to the data shown in Table S1, the OER has already been measured in eight different types of cells irradiated with X-rays and charged particles.

**Figure 6:**
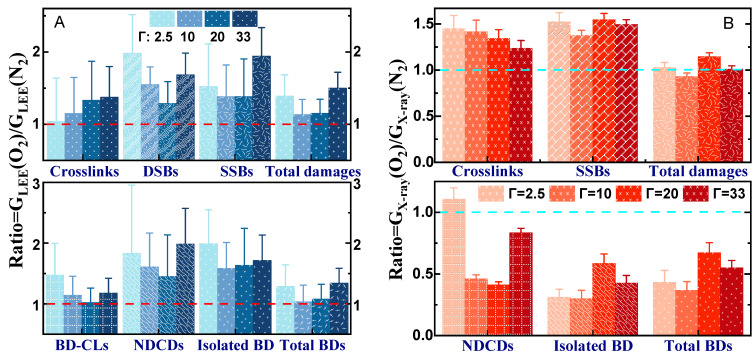
The oxygen enhancement ratio (OER) of G-values for LEEs (**A**) and X-rays (**B**). G_LEE_ and G_X_ are expressed as a ratio of the values obtained under an oxygen atmosphere to that in nitrogen. The error bars are the standard deviations obtained from the division. OERs of G_LEE_ and G_X_ for all damages are represented, except X-ray-induced DSBs and BD-CLs whose yields under an N_2_ atmosphere were too close to background levels to obtain meaningful results.

Figure 6A displays in blue the histograms of the ratios of G_LEE_ obtained in oxygen to those recorded in nitrogen for all types of damages as a function of hydration level. Each measurement of G_LEE_ in O_2_ and N_2_ arises initially from six independent measurements of the respective slopes of fluence–response curves, such as those exemplified in Figure 1. The OER for LEEs lies between 1 and 2 and is higher for SSBs, isolated BDs, and cluster lesions. The latter are known to be highly detrimental to cells, and their yields and G-values can be correlated to cell survival [16,79,80,81]. Thus, monitoring these parameters is of considerable relevance to radiotherapy [16,79]. According to Figure 6, the average OER of all cluster lesions cause by LEE-irradiation close to cellular conditions (i.e., Γ = 33) is 1.8, which is similar to the value of 1.9 averaged from the 37 OERs obtained from irradiated cells listed in Table S1. Thus, OERs in cells appear to be linked to the action of LEEs. The OERs for X-rays recorded under identical conditions are shown in Figure 6B (orange). G_X_ varies between 0.3 and 1.5, i.e., the OER of X-rays is lower than that of LEEs. At 100% humidity, the OER for cluster damage that could be measured (i.e., NDCDs) is only 0.8, i.e., a ratio much lower than the factor of 2 obtained with LEEs. Here again, the comparison suggests that out of the initial ion and SEs produced by HER, low-energy SEs are the species mostly involved in the oxygen effect.

### 2.4. Comparison with Other G-Values Measured from DNA Films Under an Oxygen Atmosphere at Different Hydration Levels

Comparisons of the present G-values with those evaluated by other groups are provided in Table S3 for LEEs, X-rays, and other types of HER incident on DNA films held under various hydration levels and O_2_ concentrations. Generally, HER produces lower G-values and a weaker dependence on humidity in O_2_ than LEEs. At Γ = 33, the present G-values of LEEs under 100% O_2_ are similar to those obtained previously in an atmosphere of 80% N_2_ + 20% O_2_ [54]. Another interesting comparison between the results of Table S3 relates to the larger G-values recorded in experiments with high film purity, i.e., from films not produced from DNA dissolved in a buffered solution that could protect DNA. In fact, irrespective of the HER source, all G-values below one in Table S3 were generated with a DNA film from an evaporated solution containing Tris-EDTA or phosphate buffer, whereas the highest G-values were usually obtained from scavenger-free solutions of as pure as possible DNA. These observations highlight the importance of the DNA immediate environment on the determination of G-values. Hence, for comparisons to be meaningful and relatable to the radiation source would require data recorded with DNA in the same immediate environment. These conditions were fulfilled in the present work, as we measured the difference between G-values for X-ray photons and LEEs at SATP, with identical film preparation and experimental conditions. Target purity in relation to G-values has recently been addressed in detail by Yu et al. [82].

### 2.5. G-Values Determined from Thin Films vs. Those Obtained in Solution

We find in the literature two main types of experiments to determine the effect of water and oxygen on DNA damage yields and hence investigate the indirect effect of HER: those performed on DNA dissolved in a water solution and those with thin DNA films surrounded by a gaseous atmosphere at different levels of humidity and oxygen. In solution, the indirect effect is probed by using radical scavengers to reduce the reactive species produced by water radiolysis with or without the presence of oxygen [83,84,85,86,87,88,89,90,91]. If all radicals created by HER outside DNA could be scavenged, a priori, the G-values arising from the direct effect could be determined, since the indirect effect would be eliminated. In this case, the DNA remains dissolved in water and is not expected to change configuration. However, considering the high concentration of scavengers needed in such measurements, they could also react with DNA, modify its reactivity, and change the properties of water. Moreover, solution experiments do not necessarily eliminate all reactive species, particularly those formed close to DNA, which could react rapidly before being scavenged. Another consideration relates to the reactions of radicals created in the first hydration layer, where water binds to DNA. It may be impossible to prevent them from reacting with DNA. All these problems are eliminated in experiments that do not use scavengers.

Film experiments performed at different hydration levels can include progressively the effect of water radiolysis, without using scavengers. However, the configuration of DNA and H_2_O-DNA and H_2_O–H_2_O interactions also change as a function of Γ, and only when bulk hydration is reached can comparisons of G-values be made with those obtained from solutions. The coordination of water molecules within and around DNA depends on their location within the hydration shell [92,93]. The hydration level Γ = 2.5, (i.e., 2.5 H_2_O molecules per nucleotide) can be considered as structural water around DNA similar to that found in a dry film under vacuum [51,94]. The addition of the water molecules extends the base-pair separation of ~2.6 Å in A-DNA to ~3.4 Å in B-DNA by introducing water–water hydrogen bonding [94]. The DNA conformation changes from the A to B form with increasing hydration level from 2.5 to 33. The B form is the biologically active DNA in chromatin. In cells, the A form can be produced during the processes of dehydration and protein binding [95].

Thus, when attempting to determine the indirect effect of LEEs from film experiments, we must consider that changes in G-values with increasing Γ can arise from modifications of DNA configuration and molecular interactions, in addition to the reaction of the species produced around DNA by LEE impacts on water and O_2_ or their combination. More generally, the indirect effect of radiation must be seen in any change in induced radiation damage to isolated DNA by its environment. Both thin film and solution experiments provide complementary information on the direct and indirect effects of HER. The thin film approach may also allow G-values to be determined under conditions closer to those of cells via the addition of biomolecules in the proportion that they are found surrounding DNA in the nucleus and mitochondria, e.g., recent work on the LEE interaction with arginine bound to plasmids [29]. Arginine is a major component of histone proteins bound to nuclear DNA to form chromatin [96].

### 2.6. Reactions of Species Produced Around DNA by LEEs Interacting with Water and O_2_

O_2_ can dissolve in water [97], and being electrophilic, it likely binds to electron-rich regions of DNA such as the N lone pairs of the bases and the ${\mathrm{PO}}_{4}^{-}$ groups of the backbone. The preferred bonding site is located at the N7 position of guanine, but the binding energy is small, i.e., of the order of *k_B_*T at 300 K [98,99,100,101]. We therefore expect that at SATP, molecular oxygen adsorbs, at least temporarily, on plasmids and surrounding water molecules forming the hydration layers. Thus, considering the equilibrium existing between condensed and gaseous oxygen, species created by X-rays propagating in our films are expected to interact with condensed-phase and gaseous molecular oxygen. Such reactive species include cations, SEs, and LEEs. SEs with energies beyond the ionization potential further create cations and LEEs. In summary, X-rays essentially produce cations and LEEs. Our experiments are designed to measure the DNA damage induced by LEEs via subtraction of the damages caused by cations or species from their reaction with oxygen or water. For comparison, we provide the G-values measured with only X-rays. The oxygen effect in DNA irradiation by HER has been investigated by many groups (Table S3) [62,63,64,65,66,67].

The 0–30 eV LEPEs in our experiment can interact with ground-state O_2_ and H_2_O to form TAs or directly scatter from these molecules. Direct scattering can lead to dissociation into neutral species, ionization, dipolar dissociation, and dissociative ionization [102,103,104]. Although a multitude of reactive species can be produced by the LEE interaction with O_2_ and H_2_O in the 0–30 eV range, we expect those in our experiments generated by LEPEs of energies below about 12 eV to be dominant for several reasons. (1) According to our LEPE distribution Z(E) shown in Figure S1, the number of photoelectrons produced by 1.5 keV X-rays incident on the Ta surface decreases inversely with electron energies from its maximum around 1.2 eV, e.g., almost 95% of emitted electrons have energies below 20 eV; (2) multiple scattering of these LEEs in the film further decreases their energies [4,105]; and (3) below 12 eV, the dominant process is TA formation [8,106]. TAs can decay via DEA, autodetachment, and resonance stabilization [12]. We discuss below principally the primary interactions of <12 eV electrons, which should constitute the main contribution to DNA damages in our experiments.

In O_2_, DEA leads to dissociation via e−+O2→O2−∗→O−(P3)+O(P3,D1) at 6, 8.4 and 13 eV [107,108,109], whereas near zero eV at SATP, the resonance stabilization channel dominates, giving rise to the three-body attachment reaction $e^{-}+O_{2}+O_{2}\to O_{2}^{-}+O_{2}$ [110]. Any production of ^3^P and ^1^D atomic oxygen below 12 eV should principally arise from the decay of TAs via autoionization that would leave the target molecule in a dissociative state [108,109]. Electron autodetachment from O_2_ TAs also leads to vibrational excitation and long-lived singlet delta states with considerable intensity [111]. Singlet-delta oxygen (^1^Δ_g_) is known from photodynamic investigations to be strongly biologically active; ^1^Δ_g_ preferentially targets guanine and oxidizes it to 8-oxo-7,8-didydroguanine [101] via both autoionization and DEA. We therefore expect considerable production of highly reactive molecular and atomic oxygen, which could damage DNA. Even the less reactive peroxyl radicals $O_{2}^{•-}$ created by resonance stabilization can further react with DNA, leading to a plethora of oxidized products, including base modifications, strand breaks, and tandem lesions [63,112]. Furthermore, within the vicinity of H_2_O, any solvated electrons produced by LEE radiolysis of bulk water at hydration level Γ = 33 could be effectively scavenged by O_2_, producing more $O_{2}^{•-}$ [21]. On the other hand, within plasmid films, caging could become effective and lower the production of atomic oxygen via dissociation of O_2_ dissociative states [35]. With bulk water around DNA, LEEs can also induce the indirect effect $e^{-}+H_{2}O\to {H_{2}O^{*-}\to OH}^{∙}+H^{-}\overset{+H_{2}O}{\to }H_{2}+{OH}^{-}+{OH}^{∙}$.

The impact of LEEs on H_2_O has been investigated in detail in the condensed phase by Orlando and co-workers [103,104,105]. The energy dependence of DEA to H_2_O is well documented, both in the gas [113] and condensed [106] phases. It occurs via the formation of core-excited resonances consisting of an (4*a*_1_)^2^ electron pair bound to a positive ion core. In the gas phase, these resonances occur at energies of 6.65 eV, 9.25 eV, and 12.75 ± 0.3 eV for the configurations ^2^*B*_1_: (1*b*_1_)^−1^(4*a*_1_)^2^, ^2^*A*_1_: (3*a*_1_)^−1^(4*a*_1_)^2^, and ^2^*B*_2_: (1*b*_2_)^−1^(4*a*_1_)^2^, respectively [113]. Each of these states generates H^−^, O^−^, and OH^−^ [113]. DEA to condensed H_2_O results principally in the formation of H¯, and the OH radical from dissociation of the ^2^B_1_ state of H_2_O¯ located in the 6−8 eV region, i.e., e^−^ + H_2_O → H_2_O^−^ → H^−^ + OH^•^ [106]. The OH radical is regarded as the most reactive oxidative species causing DNA damages [21,112]. Smaller contributions arise from the ^2^A_1_ and ^2^B_2_ anionic states, which are formed near 9 and 11 eV, respectively [106]. Collectively, these reactions lead to an abundant production of OH and H radicals and H_2_ molecules. 

Ptasińska et al. provided further details on DNA lesions induced by LEEs interacting with monolayer films of the short oligonucleotide GCAT, composed of the four DNA bases covered with three H_2_O monolayers [114]. The H_2_O film thickness corresponded to 60% of the first hydration layer. Below 15 eV, the anions H^−^, O^−^, and OH^−^ desorbed from a new type of dissociative core-excited TA formed via electron capture by a DNA-H_2_O complex. A smaller portion of the H^−^ desorption signal arose from weakly bonded H_2_O molecules. The overall anion yield from the oligonucleotide was increased by a factor of 1.6 in the presence of water [114]. This finding indicates that beyond damage caused by the active species of oxygen and water mentioned in this section, other phenomena present at the interface between DNA and its surroundings could contribute to DNA damage. In summary, apart from modification of LEE–DNA interactions by O_2_ and H_2_O, the observed increase in G_LEE_ with oxygen and hydration levels should arise principally from reactions with DNA of atomic oxygen, O^−^, peroxyl radicals $O_{2}^{•-}$, ^1^Δ_g_, and OH^•^. The G_LEE_ values are also expected to depend on the synergistic interaction of the reactive species from the LEE interaction with O_2_ and H_2_O.

### 2.7. Oxygen Fixation of LEE-Induced DNA Damage

The direct interaction of LEEs could effectively produce free radicals within DNA (DNA^•^) via DEA, which can react with O_2_, forming peroxyl radicals (DNA-$O_{2}^{•-}$) [11,112,115]. Such oxygen binding reactions have been observed to cause LEE-induced damage in alkanes and oligonucleotides [73,116]. Recently, much evidence showed that reactive peroxyl radicals can propagate damage to vicinal components in DNA strands, leading to the tandem lesions [67]. Consequently, the oxygen is “fixed” by that reaction, and the generation of non-restorable organic peroxide (DNA-OOH) can lead to DNA damage. Furthermore, large amounts of neutral hydrogen radicals are generated via DEA, even at subexcitation energies (<3 eV), from DNA components such as nucleobases and deoxyribose [117,118,119]. The subsequent reaction, $H^{∙}+O_{2}\to HO_{2}^{∙}$, leads to the formation of radical $HO_{2}^{∙}$, which could be involved in the DSB formation by oxygen radicals [120]. Since LEEs strongly interact with DNA [23], oxygen fixation is expected to occur and enhance DNA lesions [121].

## 3. Materials and Methods

In this section, we provide a brief description of the experimental procedure to measure under SATP the loss of the supercoiled plasmid configuration, SSBs, DSBs, BDs, prompt CLs, base-damage related CLs (BD-CLs), and non-DSB cluster damages (NDCDs) induced by X-rays and the LEPEs they produce. These damages are measured for X-rays incident on five monolayers of plasmid DNA deposited on glass and Ta substrates to generate the G-values from photon (G_X_) and LEE (G_LEE_) irradiations, respectively. G_LEE_ is deduced by subtracting the yield of a specific lesion recorded with the glass substrate from that measured with the plasmids deposited on Ta. The preparation of plasmid DNA, enzyme treatment, electrophoresis analysis, X-ray irradiation dosimetry, and G-value calculations were reported previously [52,56]. Details more relevant to the present investigation are provided in the Supplementary Materials.

### 3.1. Preparation of Plasmid DNA Films

Plasmid DNA (pGEM-3Zf(-), 3197 bp) is extracted from *E. coli* with a HiSpeed plasmid Maxi kit (QIAGEN). The purified plasmids in distilled deionized water consist of 96.0% supercoiled, 0.73% CLs, 0.72% SSBs, and 2.54% concatemeric forms. No salt is added throughout the procedure, e.g., no Tris-EDTA buffer is used as a stabilizer [75]. The concentration and purity of the DNA solution are monitored by a Synergy HT-I spectrophotometer via ultraviolet (UV) optical density absorption [122]. The quantity of proteins is estimated by the ratio of UV absorption ranging from 260 nm to 280 nm. This ratio is close to 2.0, implying less than 1% of proteins [123,124,125]. Five-monolayer (15 nm) DNA films on clean Ta surfaces and glass plates are prepared by lyophilization after deposition of 10 µL and 6 µL DNA solutions, respectively, each containing 320 ng of plasmids [75]. The average film thickness is estimated from the density of DNA (1.7 g/cm^3^), assuming the molecules are uniformly distributed within a circle of 2 ± 0.1 mm radius.

### 3.2. X-Ray Irradiation Under Controlled Hydration Levels and O_2_ Atmosphere

Irradiations are conducted in a home-made apparatus shown schematically in Figure S2 of the Supplementary Materials. It essentially incorporates a 1.5 keV Al K_α_ source and a chamber where multiple thin DNA films under different atmospheres at SATP can be displaced in front of the source. The entire system has been described in detail in previous papers [52,53,54,55,56]. We provide in this Section a brief description of the irradiation procedure.

The photon fluence at the film surface is measured by a radiochromatic dosimetry film (Gafchromic^TM^ Type HD-V2, Ashland Inc., Ashland, KY, USA) placed near each individual DNA sample deposited on Ta or glass substrates. The X-ray photons that traverse the DNA film condensed on Ta produce the distribution Z(E) of SEs at the interface, shown in Figure S1. Ninety-five percent of this distribution consists of electrons having energies below 30 eV, which peaks at 1.2 eV and has an average energy of 5.33 eV. It is normalized to the total number of electrons emitted from the Ta–DNA interface η_e_. The latter is calculated from the photoelectric efficiency of this interface (Supplementary Materials, Section S1) and estimated to be 0.057 ± 0.005 electrons emitted per incident photon [56].

All experiments are performed under 99.9% pure O_2_ at SATP. The humidity of the irradiation chamber is controlled by fluxing the dry oxygen through various concentrations of K_2_CO_3_ solution, while monitoring relative humidities (Γ = 2.5, 10, 20, and 33) with a hygrometer sensor (Traceable Hygrometer, Fisher Scientific, Wilmington, DE, USA). Experiments are performed at relative humidities of 0 ± 0.1%, 50 ± 2.3%, 80 ± 1.9%, and 100 ± 1.0%, respectively. For each humidity level (1) a total 36 DNA films are deposited on Ta and glass, (2) 30 DNA films are exposed to X-rays for 5, 10, 20, 30, and 40 min with six unirradiated control samples, and (3) Gafchromic^TM^ films are exposed to X-rays in situ with the DNA samples. The latter measurements allow the photon fluence at the film’s surface to be determined, irrespective of additional absorption by oxygen and water between the source and the surface. Following X-ray exposures, the respective dosimetry films are stored in darkness for 24 h at room temperature before being scanned in jpeg format with a RICOH MP C3004ex scanner. The average pixels of red and green mode channels are analyzed with ImageJ software. The mean value of color histograms is used to calculate the irradiation dose and photon fluence (photons/cm^2^) for each irradiated sample [56,126].

### 3.3. Enzyme Treatment and Analysis of DNA Damages

After irradiation, the plasmid samples are removed from the chamber and dissolved in 20 μL of distilled deionized water. The solution is divided into 4 portions to be analyzed by electrophoresis: one for immediate analysis, one to be heated at 37 °C for 1 h to reveal any heat labile effects, and two other portions to be treated, respectively, with 0.5 and 1 unit of *E. coli* base excision repair endonuclease III (Nth) and formamidopyrimidine N-glycosylase (Fpg) (Trevigen Inc., Gaithersburg, MD, USA) enzymes at 37 °C for 1 h [127,128]. SSBs, DSBs, and CLs form specific bands in the electrophoresis gel due to their circular and linear configurations or a combination of these forms, respectively, which have a different mobility. These configurations can be revealed without sample treatment. As for samples treated with enzymes, the reactions are stopped by the addition of 0.5 M EDTA buffer and kept on ice prior to further analysis. Nth and Fpg can specifically recognize and remove pyrimidine and purine bases, respectively, by hydrolysis of the glycosidic bond, to form strand breaks, detectable as circular and linear configurations via the electrophoretic assays [129,130,131]. Hence, the additional yields from these treatments correspond to those arising from pyrimidine and purine BDs, respectively. The additional SSB, DSB, inter-duplex CL, and loss of supercoiled yields from the enzyme-treated samples correspond to the yields of isolated BDs, NDCDs, BD-CLs, and total BDs, respectively.

Prior to electrophoresis analysis, the plasmid samples are stained with SYBR Green I at 100× diluted concentrations and mixed with the loading buffer. The DNA conformations are analyzed by 1.1% agarose gel electrophoresis in 1× TAE buffer (40 mM Tris acetate, 1 mM EDTA, pH 8.0) at a field strength of 5 V/cm for 10 min, followed by 4 V/cm for 90 min. A normalization factor (1.12) is applied to the yields of the supercoiled configuration to account for the weaker binding of SYBR Green I, as compared to the other configurations. The gels were scanned by a STORM860 in the blue fluorescence mode at an excitation wavelength of 430 nm and quantified by ImageQuant 5.0 (Molecular Dynamics) software.

### 3.4. Yields and G-Values of DNA Damages Induced by 1.5 keV X-Rays and LEPEs

The effective yields per photon·molecule·cm^−2^ of each damage type are recorded for DNA on glass and Ta. These yields can be obtained by fitting a linear function to their respective exposure–response curves and dividing its slope by the initial percentage of supercoiled DNA in the unirradiated sample [52,56]. According to previous analysis, any charge accumulation in the target does not influence the value of the yields in the linear regime of exposure–response curves [132]. The total yields of DNA damage and BD can also be deduced from exposure–response curves by considering the yield of heat-labile sites. Subtracting the yield of a lesion obtained with Ta (Y_Ta_) from that recorded on glass (Y_glass_) provides the yield arising from DNA damage Y_LEE_ induced by LEEs as Y_LEE_ = Y_Ta_ − Y_Glass_ [56]. This simple relationship assumes that the photoelectron current emitted from glass is negligeable compared to the one emitted from Ta. This is the case, as shown in Figure S3, from the comparison of the 1.5 keV X-ray SE distribution emitted from Ta and glass substrates.

The number of DNA damages (D) per photon·cm^−2^ arising from LEE and photon interactions can be deduced as D_LEE_ = Y_LEE_ × N_DNA_ and D_Glass_ = Y_Glass_ × N_DNA_, respectively, where N_DNA_ is the number of 3197 base pairs plasmids in each sample. However, determination of the G-value for LEPEs requires that the total energy of these electrons be deposited in the DNA film. To correct for the finite thickness of our films, the G-values measured in the present experiments were multiplied by a correction factor (CF), previously derived from the thickness dependence of LEE and X-ray G-values for the loss of the supercoiled configuration, SSBs and DSBs, in a nitrogen atmosphere at SATP [49,133]. G_X_ and G_LEE_ are calculated from Equations (1) and (2), respectively, with errors arising from film thickness and area, photon and LEPE fluence, CF, and humidity control [49,56,133].

(1) $$G_{X}=\frac{D_{Glass}}{1486(eV)X_{Abs}}\times {CF}_{X}\times 100$$

(2) $$G_{LEE}=\frac{D_{LEE}}{\eta_{e}X_{Trans}\bar{E}}\times {CF}_{LEE}\times 100$$

where *η_e_* is the total number of electrons emitted from the Ta–DNA interface. *X_Abs_* represents the number of photons absorbed in the DNA film, whereas *X_Trans_* corresponds to the number of photons passing through the film to penetrate the tantalum substrate and produce LEPEs [56,134]. The values of the correction factors for X-rays and LEPEs are *CF_X_* = 1.094 and *CF_LEE_* = 1.05, respectively [49,133].

## 4. Summary

The present results provide the first complete set of G-values (i.e., damage yields per energy deposited) for all measurable conformational damages induced to plasmid DNA by the direct and indirect effects of LEEs under an oxygen atmosphere at SATP. G-values for the same types of damage induced by 1.5 keV X-rays in the same plasmid films are also reported under identical conditions. In O_2_, all G-values for LEEs (G_LEE_) progressively increase, as a function of hydration level, by factors ranging from 1.4 for DSBs up to 2.2 for loss of supercoiled, respectively. On the contrary, G-values for X-ray irradiation (G_X_) decrease by factors of 1.4 and 1.2 for DSBs and NDCDs, respectively, and increase 1.2- and 1.5-fold for SSBs and isolated BDs, respectively. At 100% humidity, G_LEE_ is always larger than G_X_, and the ratio G_LEE_/G_X_ varies from 4 for CLs to 18 in the case of isolated base damages. The potentially lethal lesions (i.e., DSBs and NDCDs) created by LEE reach maximum G-values 10- to 15-fold larger than those produced by X-rays, respectively, separately in a 100% and 80% humidity atmosphere. The later comparison indicates that LEEs are much more efficient than 1.5 keV photons to create detrimental cellular damage in an oxygenated medium.

This study follows recent investigations performed in a nitrogen atmosphere, with the same apparatus under identical conditions. Comparison of the results from the latter experiments with the present ones allows to examine the effect of oxygen on LEE G-values for all types of lesions reported. Irrespective of hydration level, the yields of all LEE damages increase by factors varying from 1 to 2 in the presence of molecular oxygen. Apart from CLs, whose oxygen enhancement factor (OER) increases progressively from 1 to 1.4, no specific trend is observed as a function of humidity percentage. The average OER of cluster lesions for LEE irradiation close to cellular conditions (i.e., Γ = 33) is 1.8. Such lesions are mostly responsible for the decrease of cell survival after irradiation with HER. Interestingly, this OER is very close to the value of 1.9 obtained from an average of 37 biological experiments with eight different types of cells mostly irradiated with X-rays. Since most of these experiments were performed with cancer cells and patient-derived stem cells (Supplementary Materials), the comparison suggests that LEEs play an important role in oxygen radiosensitization during radiotherapy.

Ideally, measurements of LEE G-values [49,51,56] and damage cross sections [135] for DNA should, in addition to water, also incorporate an environment of biomolecules found in the cell, e.g., amino acids [29], histones, proteins, and oxygen. Such experimental data are particularly needed as input parameters in calculations that can directly use LEE cross-sections and G-values of physicochemical and chemical processes to estimate the production of different types of DNA lesions within nano-volumes of a cell and its environment [136,137,138]. These calculations should be particularly relevant to radiotherapies producing high densities of LEEs such as ion [139], flash [140,141], and Auger electron [142] radiotherapies. The latter is particularly promising in the development of radioactive compounds targeted to cancer cells and their nucleus, which have the potential to selectively destroy malignant tumors while sparing healthy tissue [143,144].

## Figures and Tables

**Table 1:** The G-values (number of a specific DNA damage/100 eV of energy deposited) for LEEs (G_LEE_) and 1.5 keV X-rays (G_X_) obtained from the experimental values multiplied by a factor CF to correct for the finite thickness of the 15 nm films. Experimental G-values were recorded at different hydration levels Γ under an O_2_ atmosphere at SATP. ER is the enhancement ratio of G-values in going from Γ = 2.5 to 33. G_HER_ represents G-values calculated for two typical LEE energy distributions produced by high energy radiation (HER). The distributions are shown in Figure S1, and the details of this type of calculation are given in the Supplementary Materials.

G-Value (D/100 eV)	Hydration Level (Γ)	CLs	SSBs	DSBs	Loss of Supercoiled	BD-CLs	Isolated BD	NDCDs	Total BDs	Total Damages
**G_LEE_** **CF = 1.050**	**2.5**	0.3 ± 0.1	7.8 ± 2.1	1.4 ± 0.3	8.3 ± 2.3	0.7 ± 0.1	12.5 ± 1.5	1.5 ± 0.2	13.1 ± 1.7	21.4 ± 2.0
	**10**	0.5 ± 0.1	9.7 ± 2.2	1.4 ± 0.1	10.0 ± 1.8	0.9 ± 0.1	14.1 ± 2.0	1.7 ± 0.1	14.8 ± 1.9	24.9 ± 2.0
	**20**	0.6 ± 0.1	11.8 ± 1.9	1.5 ± 0.1	12.7 ± 2.3	1.0 ± 0.1	17.4 ± 2.0	1.7 ± 0.1	19.2 ± 1.9	32.0 ± 2.1
	**33**	0.7 ± 0.1	16.7 ± 1.7	2.0 ± 0.1	18.0 ± 2.1	1.2 ± 0.1	19.5 ± 1.9	2.4 ± 0.1	24.5 ± 1.9	42.5 ± 2.2
**ER_LEE_**	**2.5** **→** **33**	2.1	2.1	1.4	2.2	1.7	1.6	1.6	1.9	2.0
* G_HER-1_	**2.5**	0.2 ± 0.1	3.8 ± 1.0	1.2 ± 0.2	4.0 ± 1.1	0.4 ± 0.1	4.3 ± 0.5	1.3 ± 0.2	4.6 ± 0.6	8.9 ± 0.8
**G_HER-2_**	**2.5**	0.3 ± 0.1	6.9 ± 1.9	1.4 ± 0.3	7.3 ± 2.1	0.7 ± 0.1	10.7 ± 1.3	1.5 ± 0.2	11.2 ± 1.4	18.6 ± 1.8
**G_X_** **CF = 1.094**	**2.5**	0.17 ± 0.02	3.3 ± 0.2	0.28 ± 0.03	3.5 ± 0.3	0.09 ± 0.01	0.7 ± 0.2	0.27 ± 0.02	0.8 ± 0.2	4.2 ± 0.2
	**10**	0.16 ± 0.01	3.4 ± 0.1	0.28 ± 0.01	3.7 ± 0.1	0.09 ± 0.01	0.7 ± 0.2	0.12 ± 0.01	0.9 ± 0.2	4.6 ± 0.2
	**20**	0.17 ± 0.01	4.0 ± 0.2	0.18 ± 0.01	4.0 ± 0.2	0.10 ± 0.01	1.1 ± 0.2	0.11 ± 0.01	1.4 ± 0.2	5.4 ± 0.2
	**33**	0.16 ± 0.01	3.9 ± 0.1	0.20 ± 0.01	3.9 ± 0.2	0.09 ± 0.01	1.1 ± 0.2	0.22 ± 0.01	1.5 ± 0.2	5.5 ± 0.2
**ER_X_**	**2.5** **→** **33**	1.0	1.2	0.7	1.1	1.1	1.5	0.8	2.0	1.3

The types of damages, G-values, their ratios (ER) at Γ = 33 vs. 2.5 and the hydration level Γ are indicated in bold characters. * HER-1: G-value calculated from the energy distribution of electrons created by the initial ionization. HER-2: G-value calculated from the energy distribution of all secondary electrons.

## Data Availability

The data presented in this study are available on request from the corresponding author.

## Supplementary Materials

The following supporting information can be downloaded at https://www.mdpi.com/article/10.3390/molecules29246033/s1: Figure S1: Three types of LEE distributions within the range 0–20 eV; Table S1: Oxygen enhancement ratio (OER) induced by HER in cells; Figure S2: Schematic diagram of the apparatus used to irradiate plasmid DNA samples with 1.5 keV Al K_α_ X-ray photons under oxygen held at SATP; Table S2: Measured G-values (i.e., number of a specific DNA damage (D) per 100 eV of deposited energy) for LEEs (G_mLEE_) and 1.5 keV X-rays (G_mX_). G_mLEE_ and G_mX_ were recorded at different hydration levels (Γ = 2.5 to 33) under an O_2_ atmosphere at SATP; Figure S3: SE emission from Ta, glass, and glass circumscribed with Ag gels, induced by 1.5 keV X-rays; Table S3: The first eight lines provide the present G-values for various DNA lesions induced in plasmid films by LEEs and 1.5 keV X-rays in an O_2_ atmosphere at SATP, under different hydration levels. These are compared to G-values resulting from exposure of DNA films to similar and other ionizing radiation sources under a controlled O_2_ atmosphere or in air. Refs. [3,4,16,54,56,48,49,52,82,83,133,145,146,147,148,149,150,151,152,153,154,155,156,157,158,159,160,161,162,163,164,165,166,167] were cited in Supplementary Materials.

## Abbreviations

BD	base damage
BD-CL	base-damage related CL
CF	correction factor
CL	crosslink
DEA	dissociative electron attachment
DSB	double strand break
Fpg	formamidopyrimidine N-glycosylase
HER	high-energy radiation
LEE	low-energy electron
LEPE	low-energy photoelectron
NDCD	non-DSB cluster damage
Nth	endonuclease III
OER	oxygen enhancement ratio
SATP	standard atmospheric temperature and pressure
SE	secondary electron
SSB	single strand break
Ta	tantalum
TA	transient anion
