# Supplementary material for: Oxygen Effect on 0–30 eV Electron Damage to DNA Under Different Hydration Levels: Base and Clustered Lesions, Strand Breaks and Crosslinks
Source: Molecules. 2024 Dec 21;29(24):6033. doi: 10.3390/molecules29246033 (PMC11680046; doi:10.3390/molecules29246033)
Supplement: Supplementary file 1 [file molecules-29-06033-s001.zip › molecules-3306263-supplementary.pdf]

Supporting Information

Oxygen Effect on 0–30 eV Electron Damage to DNA Under Different Hydration Levels: Base and Clustered Lesions, Strand Breaks and Crosslinks

Yingxia Gao [1], Xuran Wang [1], Pierre Cloutier [2], Yi Zheng [1,2] and Léon Sanche [2,*]

[1] State Key Laboratory of Photocatalysis on Energy and Environment, Fuzhou University, Fuzhou 350116, China; gyxmyr3719@163.com (Y.G.); xr_wang1215@163.com (X.W.); yi.zheng@usherbrooke.ca (Y.Z.)

[2] Department of Nuclear Medicine and Radiobiology and Clinical Research Center, Faculty of Medicine and Health Sciences, Université de Sherbrooke, Sherbrooke, QC J1H 5N4, Canada ; pierre.cloutier@usherbrooke.ca

* Correspondence: leon.sanche@usherbrooke.ca

1. Production of Secondary Electrons (SEs) from the Ta-DNA Interface.

The yield $\eta(E_k)$ of slow SEs resulting from X-ray impact on a metal is obtained from the equation

$$\eta(E_k) = \eta_s E_k/(E_k + W)^4 \qquad \text{(S1)}$$

where E_k is the electron kinetic energy, η_s the yield normalization coefficient, and W the work function [1]. With W = 3.7 eV for the polycrystalline tantalum (Ta) substrate covered with DNA measured by a Kelvin probe [2], we get the typical energy distribution of SEs emitted from Ta induced by 1.5 KeV X-rays in the range of ≤ 20 eV shown in blue (Z) in Figure S1. Its intensity inversely decreases with electron energy after a peak around 1.2 eV. From this distribution, we determine the mean electron energy to be 5.33 eV. The current of photoelectrons emitted from a bare Ta substrate of work function W = 4.22 eV was reported by Alizadeh et al. from which the photelectric efficiency of their Ta surface was estimated to be 0.047 ± 0.005 electrons emitted per photon [3]. By normalizing the two distributions (calculated with W = 3.7 eV for the DNA-Ta interface of the present work and W = 4.22 eV from Alizadeh et al.) so that they coincide above 15 eV, and by comparing the relative total intensities, the photoelectric efficiency of the Ta-DNA interface was estimated to be 0.057 ± 0.005 electrons emitted per photon. This value is used to calculate the G-values for LEEs in present work.

Our previous paper has showed that once a G-value is obtained in a given atmosphere for a specific photoelectron energy distribution, it can be calculated for another such distribution, as long as the electron energy loss function is known in the range of interest [4]. For 0-20 eV electrons, this function is known for dry DNA [5]. We therefore provide, in Table 1 of the main text, the G-values for 0-20 eV electrons hitting a dry DNA film in oxygen with the two other distributions shown in Figure S1.

2. Measurement of secondary electron spectrum.

The secondary electron (SE) distributions from Ta and glass were independently characterized by X-ray photoelectron spectroscopy (XPS), using the commercial system Thermo

Scientific ESCALAB250Xi located at Fuzhou University. The XPS electron energy analyzer cannot adequately measure the magnitude SE spectrum at energies below 7 eV due to the energy cutoff of the electrostatic lenses and deflector. The comparisons of the energy distributions can be made from the higher-energy portion of the electron energy-loss spectra. The present XPS is operated at the base pressure of 5×10^{-10} mbar. The X-ray source consists of monochromatized Al K_α photons ($h\nu$=1486.68 eV, R=500 μm). Ta and 2 pieces of glass (5×5 mm) are fixed on the metal sample holder with conductive glues. One of the glass plates was circumscribed with a silver gel (SINWE 3703) to avoid charging of the surface [6]. The silver glue solvent was evaporated in a silicon-filled drying tower before transfer to the XPS chamber.

The SE emission measurements were performed in the LEE mode with a pass energy of 0.8 eV and energy steps of 0.05 eV without the neutralizing gun. To obtain a good signal-noise ratio, the number of scans was set to 10. During the measurements, the sample platform of the XPS was connected to ground. Figure S3 shows one SE spectra from Ta and two from glass, with enlargements of the latter in the insert. The emission of SEs from Ta by X-rays are at least two orders of magnitude larger than that from glass. The results provide experimental evidence that SE emission from Ta is much larger than that from glass, i.e., SE emission from glass is negligible in our experiments. Hence, glass can be used as a substrate to measure DNA damage induced by 1.5 keV X-ray photons.

3. G Values of DNA Damages Induced by any SE distributions.

Fig. S1 shows in blue the photoelectron energy distribution Z(E) emitted from the Ta-DNA interface by incident 1.5 keV X-rays. Z(E) is derived from Eq. S1, with the yield normalization coefficient η_s = 101.5 and W the work function at the metal-DNA interface of 3.7 eV [1,2]. The initial electron energy distribution Z_{HER-1} produced by high energy radiation (HER) in water has a most probable energy of ~10 eV (orange curve in Fig. S1) [7]. Considering all electrons from ionization of water by HER, Cobut et al. [8], calculated the distribution Z_{HER-2} shown in green in Fig. S1. To obtain the G-values of all measured DNA damages for LEE distributions Z_{HER-1} and Z_{HER-2}, or others, from

that induced by our X-ray photoelectron (*Z(E)*), we can transform mathematically the G_{LEE} generated in the present work to those corresponding to other LEE distributions, such as Z_{HER-1} and Z_{HER-2} produced by HER. The model is based on measurements of the 0-20 eV electron energy dependence of effective yields *Y(E)* for various damages measured with the same plasmid DNA as that used in the present study, under the same hydration conditions (i.e., with Γ=2.5) [5].

Mathematically, the proportionality between G_{LEE}, the X-ray photoelectron distribution Z(E) and *Y(E)* can be written as $G_{LEE} \propto \frac{\int_0^{20} Y(E)Z(E)dE}{\int_0^{20} E\cdot Z(E)dE}$. Similarly, the proportionality between G_{HER}, *Y(E)* and the distribution Z_{HER} can be expressed as $G_{HER} \propto \frac{\int_0^{20} Y(E)Z_{HER}dE}{\int_0^{20} E\cdot Z_{HER}dE}$. Thus, the calculated ratio G_{HER}/G_{LEE} can convert G_{LEE} to G_{HER} by integrating the yields of DNA damages for electron energy distributions Z_{HER-1} and Z_{HER-2} between 1-20 eV. Since the *Y(E)* are recorded with the monoenergetic LEE bombardment in vacuum [5], *f* values apply to dry DNA films. The respective two factors *f* correlated for all types of DNA damage are provided in Table S2. In summary, G values G_Z for any type of DNA damage from any LEE distributions (Z) can be obtained as, $\boldsymbol{G_z = f_z \times G_{LEE}}$.

Table S1 Oxygen enhancement ratio (OER) induced by high energy radiation (HER) in cells.

Radiation			Cell	OER
X rays [9]		270 kV	Chinese Hamster V79-171 Cells	2.36(0.19)-2.87(0.16)
X rays [10]		270 kV, 15 mA	Chinese Hamster V79-753B lung Cells	3.0
X-rays (240 kVp) [11]		2.5 Gy/min,	feet skin cells of WHT mice	2.2
X-rays (200 kV, 20mA) [12]		9.4 keV/μm	squamous cell carcinoma (SCCVII) cells	1.87(0.13)-2.52(0.11)
$^{12}C^{6+}$ (290 MeV/nucleon)		46 keV/μm		1.43(0.19)-1.84(0.12)
		80 keV/μm		1.52(0.10)-1.94(0.10)
X-rays (200 kV, 20mA) [13]		~3 Gy/min	squamous cell carcinoma (SCCVII) cells	1.73(0.05)-2.58(0.09)
290 MeV/nucleon carbon		14 keV/μm		1.21(0.08)-2.55(0.15)
400 MeV/ nucleon neon		31 keV/μm		1.42(0.09)-3.25(0.16)
490 MeV/nucleon silicon		31 keV/μm		1.28(0.10)-2.99(0.17)
500 MeV/nucleon argon		89 keV/μm		1.58(0.23)-1.95(0.24)
500 MeV/nucleon iron ions		192 keV/μm		1.12(0.32)-1.19(0.20)
X-rays (225 kV, 13 mA) [14]		2 Gy/min	U251-MG glioblastoma cells	1.2±0.1
C ions		28 keV/μm		1.0±0.0
X-rays (225 kV, 13 mA)		2 Gy/min	GL15 glioblastoma cells	1.3±0.2
C ions		28 keV/μm		1.4±0.1
		50 keV/μm		1.3±0.2
		100 keV/μm		1.0±0.1
Proton [15]		155 MeV/n	human salivary gland tumor (HSG) cells	2.71±0.11 (AVE)
X-rays [16] (150 kVp, ≥1.82 Gy/min)		uniform field	human prostate cancer (DU145) cells	2.38±0.22
		in-field		2.26±0.25
		uniform field	non-small cell lung cancer (H1299) cells	2.30±0.27
		in-field		2.27±0.38
X-rays [17] (150 kVp, 1.82 Gy/min)		uniform field	human prostate cancer (DU145) cells	2.31
		in-field		2.31
		uniform field	non-small cell lung cancer (H1299) cells	2.31
		in-field		2.31
		uniform field	non-small cell lung cancer (H460) cells	1.32
		in-field		1.32
X-rays [18] (225 kVp)		0.59 Gy/min	normal human skin fibroblasts AG01522B cells	1.80±0.09 (D_{10})<br>2.03±0.10 (D_{50})<br>2.16±0.10 (D_{90})
			patient-derived glioblastoma stem like cells (E2 cells)	1.84±0.09 (D_{10})<br>2.24±0.11 (D_{50})<br>2.50±0.12 (D_{90})
X-rays [19] (100 kVp, 5.0 mA)		1.37 Gy/min	MDA-MB-231 breast cancer cells	1.4±0.6(clonogenic)<br>1.9±0.4(metabolic)
Grenz rays [20] (soft X-rays)	Monoenergetic 15 keV	2.7 keV/μm	Monte Carlo Damage Simulation (MCDS) for cell survival	2.5
	Monoenergetic 10 keV	3.7 keV/μm		2.4
	10 kV (ave:5.7 keV)	5.6 keV/μm		2.1
	4.6 kV (ave:4.55 keV)	6.6 keV/μm		2.0
^{60}Co γ-rays	1250 kV	0.24 keV/μm		2.8

Table S2 Measured G-values (i.e., number of a specific DNA damage (D) per 100 eV of deposited energy) for LEEs (G_{mLEE}) and 1.5 keV X-rays (G_{mX}). G_{mLEE} and G_{mX} were recorded at different hydration levels (Γ=2.5 to 33) under an O_2 atmosphere at standard atmospheric temperature and pressure (SATP).

G value (D/100eV)	hydration level (Γ)	CLs	SSBs	DSBs	Loss of supercoiled	BD-related CLs	Isolated BD	Non-DSB Cluster damages	Total BDs	Total damages
G_{mLEE}	2.5 / dry DNA	0.3±0.1	7.4±2.0	1.3±0.2	7.9±2.2	0.7±0.1	11.9±1.4	1.4±0.2	12.5±1.6	20.4±1.9
	10 / first hydration level	0.4±0.1	9.3±2.0	1.3±0.1	9.6±1.7	0.8±0.1	13.4±1.9	1.6±0.1	14.1±1.8	23.7±1.9
	20 / second hydration level	0.6±0.1	11.2±1.8	1.4±0.1	12.1±2.2	1.0±0.1	16.6±1.9	1.7±0.1	18.3±1.8	30.4±2.0
	33 / bulk water	0.6±0.1	15.9±1.6	1.9±0.1	17.1±2.0	1.1±0.1	18.6±1.8	2.3±0.1	23.3±1.8	40.4±2.1
G_{mX}	2.5	0.15±0.01	3.1±0.2	0.25±0.02	3.2±0.2	0.08±0.01	0.6±0.1	0.25±0.02	0.7±0.2	3.9±0.2
	10	0.15±0.01	3.1±0.1	0.25±0.01	3.4±0.1	0.08±0.01	0.7±0.2	0.11±0.01	0.8±0.2	4.2±0.2
	20	0.15±0.01	3.6±0.2	0.17±0.01	3.7±0.2	0.09±0.01	1.0±0.1	0.10±0.01	1.3±0.2	4.9±0.2
	33	0.15±0.01	3.5±0.1	0.19±0.01	3.6±0.2	0.09±0.01	1.0±0.1	0.20±0.01	1.4±0.2	5.0±0.2
calibration factor	$f_{HER\text{-}1}$	0.494	0.489	0.873	0.480	0.502	0.344	0.841	0.351	0.416
	$f_{HER\text{-}2}$	0.899	0.884	1.007	0.882	0.912	0.855	1.001	0.857	0.869

CL: crosslinks, SSBs: single-strand breaks, DSBs: double-strand breaks, BD: base damage.

Table S3 The first eight lines provide the present G values for various DNA lesions induced in plasmid films by LEEs and 1.5 keV X-rays in an O_2 atmosphere at SATP, under different hydration levels. These are compared to G-values resulting from exposure of DNA films to similar and other ionizing radiation sources under a controlled O_2 atmosphere or in air. G-values are recalculated in units of number of damages/100 eV. The corresponding linear energy transfer (LET) is given when available. The experiments are generally conducted at room temperature unless otherwise indicated in degree Kelvin (K).

Radiation	Target	Humidity	CLs	SSBs	DSBs	Loss of supercoiled	Isolated BD	NDCDs
LEEs from 1.5 keV X-rays (present results)	Plasmid DNA, 3197 bp[a] (no buffer)	Γ=2.5, O_2	0.3±0.1	7.8±2.1	1.4±0.3	8.3±2.3	12.5±1.5	1.5±0.2
		Γ=10, O_2	0.5±0.1	9.7±2.2	1.4±0.1	10.0±1.8	14.1±2.0	1.7±0.1
		Γ=20, O_2	0.6±0.1	11.8±1.9	1.5±0.1	12.7±2.3	17.4±2.0	1.7±0.1
		Γ=33, O_2	0.7±0.1	16.7±1.7	2.0±0.1	18.0±2.1	19.5±1.9	2.4±0.1
1.5 keV X-rays (present results) 0.2 keV/µm	Plasmid DNA, 3197 bp (no buffer)	Γ=2.5, O_2	0.17±0.02	3.3±0.2	0.28±0.03	3.5±0.3	0.7±0.2	0.27±0.02
		Γ=10, O_2	0.16±0.01	3.4±0.1	0.28±0.01	3.7±0.1	0.7±0.2	0.12±0.01
		Γ=20, O_2	0.17±0.01	4.0±0.2	0.18±0.01	4.0±0.2	1.1±0.2	0.11±0.01
		Γ=33, O_2	0.16±0.01	3.9±0.1	0.20±0.01	3.9±0.2	1.1±0.2	0.22±0.01
LEEs from 1.5 keV X-rays [3,21]	Plasmid DNA, 3197 bp (no buffer)	Γ=2.5, O_2				4.6±0.4		
LEEs from 1.5 keV X-rays [22]		Γ=2.5, O_2		4.2±1.1	0.20±0.05	4.6±1.2		
		Γ=33, O_2		54.1±14.1	2.4±0.6	55.6±14.4		
		Γ=2.5, 20%O_2+80%N_2		2.0±0.5	0.10±0.03	2.8±0.7		
		Γ=33, 20%O_2+80%N_2		18.0±4.7	0.30±0.08	18.0±4.7		
LEEs from 1.5 keV X-rays [23]		Γ=15, air				6.7±2.2		
1.5 keV X-rays [3,24]	Plasmid DNA, 3197 bp (no buffer)	Γ=2.5, O_2				1.8±0.1		
1.5 keV X-rays [22]		Γ=2.5, O_2		1.7±0.3	0.08±0.02	1.8±0.4		
		Γ=33, O_2		7.3±1.5	0.35±0.07	7.9±1.6		
		Γ=2.5, 20%O_2+80%N_2		1.3±0.3	0.08±0.02	1.4±0.3		
		Γ=33, 20%O_2+80%N_2		2.9±0.6	0.08±0.02	3.2±0.6		
1.5 keV X-rays [23,24]		Γ=15, air				0.61±0.09		
X rays [25] 70kV 20 mA 0.0015 keV/µm	Plasmid, pUC18, 2686 bp, TE[b] buffer	Γ=2.5, air		0.67±0.07	0.05±0.01		1.2±0.2	0.03±0.02
		Γ=7.5, air		0.56±0.06	0.04±0.01		0.7±0.2	0.04±0.02
		Γ=11.5, air		0.58±0.06	0.04±0.01		0.6±0.2	0.04±0.02
		Γ=15, air		0.61±0.05	0.04±0.01		0.5±0.2	0.03±0.02
		Γ=22.5, air		0.52±0.05	0.03±0.01		0.3±0.2	0.06±0.01
X rays [26] 70kV 20 mA		Γ=2.5, air		1.20±0.04				
		Γ=22.5, air		1.77-1.99				
X rays [27]	Plasmid pEC 10 810 bp	Γ=22, air, 4K		0.8±0.1	0.10±0.01			
		Γ=22, air		0.9±0.1	0.10±0.01			

70kV 20 mA		DNA, TE buffer, pUC18 2686 bp	Γ=22, air, 4K	1.2±0.1	0.06±0.01		
			Γ=22, air	1.4±0.1	0.07±0.01		
X-rays [28]	160 kV 3 mA	Plasmid, pUC18, 2686 bp, phosphate	Γ=34.5, air	6.3±1.9	0.5±0.1		
X-rays [29]	2 keV/μm	Plasmid, pUC18, 2686 bp, TE buffer	Γ=34.5, air	27.6±5.7	0.15±0.08	76.6±9.7	0.24±0.09
H^+ [30] 10 MeV	6.39keV/μm	Plasmid pBR322, 4361 bp, TE buffer	Γ=2.5, air	1.4±0.1	0.04±0.01	1.2±0.8	0.01±0.03
20 MeV	3.64 keV/μm			0.79±0.003	0.02±0.01	1.3±0.4	0.09±0.05
30 MeV	2.61 keV/μm			0.66±0.09	0.01±0.004	1.3±0.5	0.06±0.05
^{60}Co γ-rays [30]	6 keV/μm	Plasmid, pBR322, 4361 bp, TE buffer	Γ=2.5, air	0.68±0.10	0.03±0.01	2.3±0.3	0.15±0.04
^{60}Co γ-rays [31] (92 Gy/min)		Plasmid DNA, pUC18, 2686 bp, TE buffer	Γ=4, dry air	0.52±0.09	0.05±0.005	0.8±0.3	0.06±0.03
			Γ=8, air	0.63±0.08	0.05±0.003	1.2±0.3	0.12±0.05
			Γ=14.5, air	0.67±0.05	0.05±0.011	1.2±0.3	0.12±0.06
			Γ=24.5, air	0.69±0.06	0.06±0.005	1.4±0.3	0.18±0.02
			Γ=34.5, air	0.70±0.05	0.07±0.007	1.9±0.3	0.21±0.05
α-particle [32] (20 Gy/min)			Γ=4, dry air	0.47±0.04	0.10±0.02	0.17±0.04	0.03±0.01
			Γ=14.5, air	0.45±0.06	0.15±0.01	0.36±0.04	0.03±0.01
			Γ=34.5, air	0.58±0.09	0.17±0.01	0.24±0.06	0.05±0.01
$^{4}He^{2+}$ [29]	82 keV/μm	pUC18, TE buffer	Γ=34.5, air	10.9	0.21	10.6	0.10
$^{4}He^{2+}$ [33]	19 keV/μm	Plasmid DNA, pUC18, 2686 bp, TE buffer	Γ=34.5, air	0.67±0.10	0.04±0.02	2.5±0.5	0.09±0.04
	63 keV/μm			0.70±0.02	0.07±0.01	1.3±0.5	0.09±0.04
	95 keV/μm			0.65±0.03	0.11±0.002	0.71±0.16	0.06±0.04
	121 keV/μm			0.47±0.05	0.04±0.02	0.60±0.19	0.05±0.05
	148 keV/μm			0.37±0.03	0.09±0.01	0.18±0.05	0.03±0.02
$^{4}He^{2+}$ [34]	2.2 keV/μm		Γ=34.5, air	0.86±0.19	0.07±0.02	2.1±1.2	0.06±0.02
	6 keV/μm			0.96±0.18	0.05±0.02	2.5±1.2	0.10±0.04
$^{12}C^{5+,6+}$ [34]	13 keV/μm	Plasmid DNA, pUC18, 2686 bp, TE buffer	Γ=34.5, air	1.6±0.4	0.12±0.05	3.8±1.8	0.13±0.05
	87 keV/μm			0.60±0.05	0.04±0.01	0.8±0.2	0.05±0.02
	122 keV/μm			0.55±0.05	0.05±0.01	1.1±0.3	0.06±0.02
	342 keV/μm			0.45±0.06	0.08±0.01	0.7±0.3	0.02±0.01
	507 keV/μm			0.49±0.05	0.08±0.01	0.5±0.2	0.01±0.01
$^{20}Ne^{10+}$ [34]	31 keV/μm	Plasmid DNA, pUC18, 2686 bp, TE buffer	Γ=34.5, air	1.4±0.3	0.08±0.02	2.7±1.9	0.08±0.03
	361 keV/μm			0.56±0.05	0.11±0.02	1.0±0.4	0.04±0.02
	491 keV/μm			0.47±0.01	0.11±0.01	1.0±0.4	0.03±0.01
	842 keV/μm			0.46±0.07	0.11±0.01	0.5±0.2	0.02±0.02

[a] bp: base pair; [b] TE: Tris-EDTA

Figure S1 Three types of LEE distributions within the range 0-20 eV. Z(E) (blue) is the X-rays-induced photoelectron energy spectrum from a Ta-DNA interface. Z_{HER-1} (orange) is the initial SE distribution created by a 1MeV ^{1}H ion [7], and Z_{HER-2} (green), the distribution of all SEs induced by a primary 20 keV electron [8]. The vertical scale corresponds to the electron density in Z(E), whereas the other two curves are drawn in arbitrary units for better visibility.

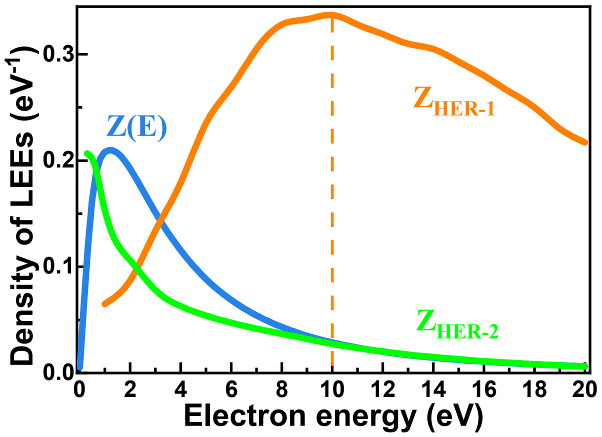

Figure S2 Schematic diagram of the apparatus used to irradiate plasmid DNA samples with 1.5 keV Al Kα X-ray photons under oxygen held at SATP: (A) baratron, (B) adjustable leak valve, (C) concave aluminum cathode, (D) high voltage electric feedthrough, (E) glass-ceramic (Macor) support, (F) quartz tube, (G) aluminum foil target, (H) He-filled enclosed volume, (I) thin foil of Mylar, (J) aluminum plate as the sample holder, (K) rotating disk, (L) gas circulation valves, and (M) humidity control by salt solutions. Further details can be found in ref. 3 and 4.

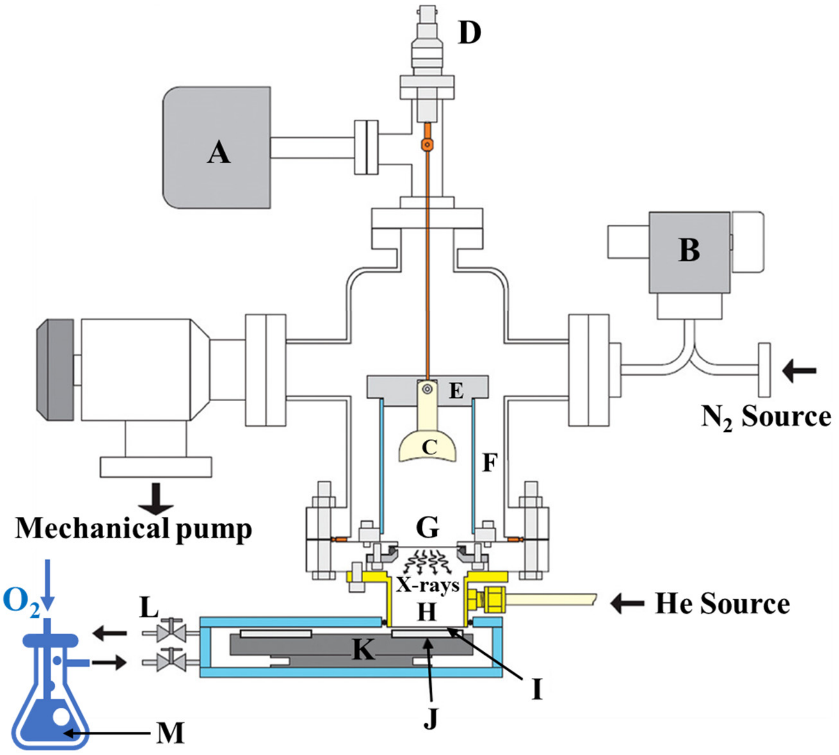

Figure S3 SE emission from Ta, glass, and glass circumscribed with Ag gels, induced by 1.5 keV X-rays. The latter two are enlarged in the insert. The decrease in intensity below 5 eV is caused by the low-energy cutoff of the electron spectrometer.

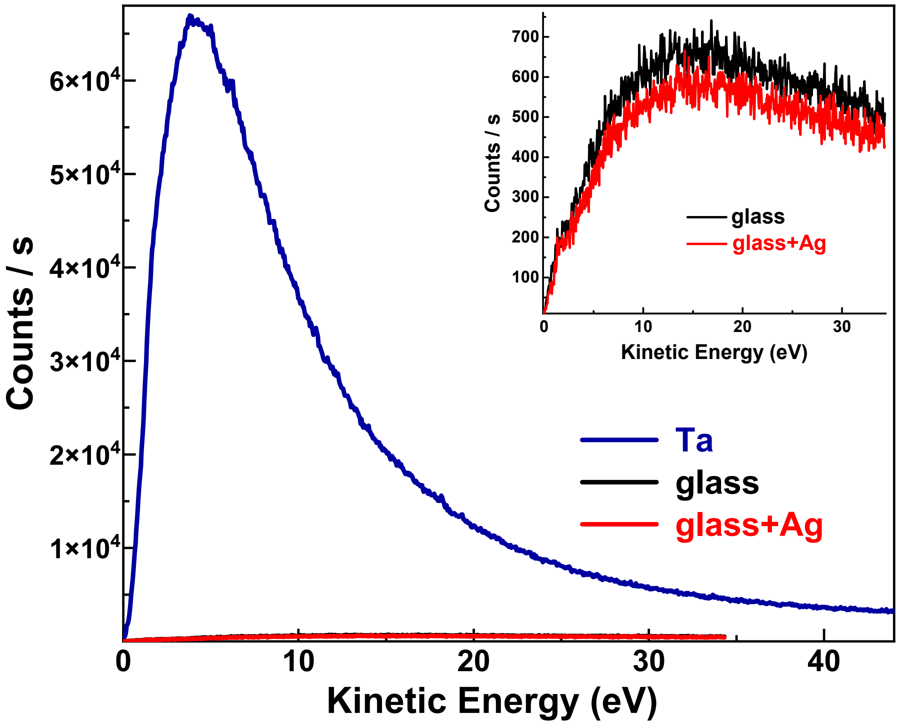

References

1. Henke, B.L.; Smith, J.A.; Attwood, D.T. 0.1-10 keV X-ray-Induced Electron Emissions from Solids—Models and Secondary Electron Measurements. *J. Appl. Phys.* **1977**, *48*, 1852-1866.
2. Liu, J.; Yao, X.; Cloutier, P.; Zheng, Y.; Sanche, L. DNA Strand Breaks Induced by 0–1.5 eV UV Photoelectrons under Atmospheric Pressure. *J. Phys. Chem. C* **2016**, *120*, 487-495.
3. Alizadeh, E.; Cloutier, P.; Hunting, D.; Sanche, L. Soft X-ray and Low Energy Electron-Induced Damage to DNA under N_2 and O_2 Atmospheres. *J. Phys. Chem. B* **2011**, *115*, 4523-4531.
4. Gao, Y.; Dong, Y.; Wang, X.; Su, W.; Cloutier, P.; Zheng, Y.; Sanche, L. Comparisons between the Direct and Indirect Effect of 1.5 KeV X-rays and 0-30 eV Electrons on DNA: Base Lesions, Stand Breaks, Cross-Links and Cluster Damages. *J. Phys. Chem. B* **2024**, *128*, 11041-11053.
5. Dong, Y.; Gao, Y.; Liu, W.; Gao, T.; Zheng, Y.; Sanche, L. Clustered DNA Damage Induced by 2-20 eV Electrons and Transient Anions: General Mechanism and Correlation to Cell Death. *J. Phys. Chem. Lett.* **2019**, *10*, 2985-2990.
6. Xiao, F.; Zheng, Y.; Cloutier, P.; He, Y.; Hunting, D.; Sanche, L. On the Role of Low-Energy Electrons in the Radiosensitization of DNA by Gold Nanoparticles. *Nanotechnology* **2011**, *22*, 465101.
7. Pimblott, S. M.; La Verne, J. A. Production of Low-Energy Electrons by Ionizing Radiation. *Radiat. Phys. Chem.* **2007,** *76,* 1244-1247.
8. Cobut, V.; Frongillo, Y.; Patau, J. P.; Goulet, T.; Fraser, M. J.; Jay-Gerin, J. P. Monte Carlo Simulation of Fast Electron and Proton Tracks in Liquid Water—I. Physical and Physicochemical Aspects. *Radiat. Phys. Chem.* **1998,** *51,* 229-243.
9. Skarsgard, L.D.; Harrison, I. Dose Dependence of the Oxygen Enhancement Ratio (OER) in Radiation Inactivation of Chinese Hamster V79-171 Cells. *Radiat. Res.* **1991**, *127*, 243-247.
10. Sapora, O.; Barone, F.; Belli, M.; Maggi, A.; Quintiliani, M.; Tabocchini, M.A. Relationships Between Cell Killing, Mutation Induction and DNA Damage in X-irradiated V79 Cells: The Influence of Oxygen and DMSO. *Int. J. Radiat. Biol.* **1991**, *60*, 467-482.
11. Stevens, G.; Joiner, M.; Joiner, B.; Johns, H.; Denekamp, J. Radiosensitization of Mouse Skin by Oxygen and Depletion of Glutathione. *Int. J. Radiat. Oncol., Biol., Phys.* **1995**, *33*, 399-408.
12. Hirayama, R.; Uzawa, A.; Takase, N.; Matsumoto, Y.; Noguchi, M.; Koda, K.; Ozaki, M.; Yamashita, K.; Li, H.; Kase, Y.; et al. Evaluation of SCCVII Tumor Cell Survival in Clamped and Non-Clamped Solid Tumors Exposed to Carbon-Ion Beams in Comparison to X-rays. *Mutat. Res., Genet. Toxicol. Environ. Mutagen.* **2013**, *756*, 146-151.
13. Hirayama, R.; Uzawa, A.; Obara, M.; Takase, N.; Koda, K.; Ozaki, M.; Noguchi, M.; Matsumoto, Y.; Li, H.; Yamashita, K.; et al. Determination of the Relative Biological Effectiveness and Oxygen Enhancement Ratio for Micronuclei Formation Using High-LET Radiation in Solid Tumor Cells: An in Vitro and in Vivo Study. *Mutat. Res., Genet. Toxicol. Environ. Mutagen.* **2015**, *793*, 41-47.
14. Valable, S.; Gérault, A.N.; Lambert, G.; Leblond, M.M.; Anfray, C.; Toutain, J.; Bordji, K.; Petit, E.; Bernaudin,

M.; Pérès, E.A. Impact of Hypoxia on Carbon Ion Therapy in Glioblastoma Cells: Modulation by LET and Hypoxia-Dependent Genes. *Cancers* **2020**, *12*, 2019.
15. Kanemoto, A.; Hirayama, R.; Moritake, T.; Furusawa, Y.; Sun, L.; Sakae, T.; Kuno, A.; Terunuma, T.; Yasuoka, K.; Mori, Y.; et al. RBE and OER within the Spread-Out Bragg Peak for Proton Beam Therapy: In Vitro Study at the Proton Medical Research Center at the University of Tsukuba. *J. Radiat. Res.* **2014**, *55*, 1028-1032.
16. Matsuya, Y.; McMahon, S.J.; Butterworth, K.T.; Naijo, S.; Nara, I.; Yachi, Y.; Saga, R.; Ishikawa, M.; Sato, T.; Date, H.; et al. Oxygen Enhancement Ratios of Cancer Cells after Exposure to Intensity Modulated X-ray Fields: DNA Damage and Cell Survival. *Phys. Med. Biol.* **2021**, *66*, 075014.
17. Matsuya, Y.; McMahon, S.J.; Butterworth, K.T.; Yachi, Y.; Saga, R.; Sato, T.; Prise, K.M. Modelling Oxygen Effects on the In- and Out-of-Field Radiosensitivity of Cells Exposed to Intensity-Modulated Radiation Fields. *Phys. Med. Biol.* **2023**, *68*, 095008.
18. Chaudhary, P.; Gwynne, D.C.; Odlozilik, B.; McMurray, A.; Milluzzo, G.; Maiorino, C.; Doria, D.; Ahmed, H.; Romagnani, L.; Alejo, A.; et al. Development of a Portable Hypoxia Chamber for Ultra-High Dose Rate Laser-Driven Proton Radiobiology Applications. *Radiat. Oncol.* **2022**, *17*, 77.
19. Carlson, N.; House, C.D.; Tambasco, M. Toward a Transportable Cell Culture Platform for Evaluating Radiotherapy Dose Modifying Factors. *Int. J. Mol. Sci.* **2023**, *24*, 15953.
20. Chan, C.-C.; Chen, F.-H.; Hsueh, K.-L.; Hsiao, Y.-Y. The Effect of Hypoxia on Relative Biological Effectiveness and Oxygen Enhancement Ratio for Cells Irradiated with Grenz Rays. *Cancers* **2022**, *14*, 1262.
21. Alizadeh, E.; Sanche, L. Measurements of G Values for DNA Damage Induced by Low-Energy Electrons. *J. Phys. Chem. B* **2011**, *115*, 14852-14858.
22. Alizadeh, E.; Sanche, L. Role of Humidity and Oxygen Level on Damage to DNA Induced by Soft X-rays and Low-Energy Electrons. *J. Phys. Chem. C* **2013**, *117*, 22445-22453.
23. Brun, É.; Cloutier, P.; Sicard-Roselli, C.; Fromm, M.; Sanche, L. Damage Induced to DNA by Low-Energy (0-30 eV) Electrons under Vacuum and Atmospheric Conditions. *J. Phys. Chem. B* **2009**, *113*, 10008-10013.
24. Alizadeh, E.; Sanche, L. Absolute Measurements of Radiation Damage in Nanometer-Thick Films. *Radiat. Prot. Dosim.* **2012**, *151*, 591-599.
25. Purkayastha, S.; Milligan, J.R.; Bernhard, W.A. On the Chemical Yield of Base Lesions, Strand Breaks, and Clustered Damage Generated in Plasmid DNA by the Direct Effect of X Rays. *Radiat. Res.* **2007**, *168*, 357-366.
26. Sharma, K.K.K.; Milligan, J.R.; Bernhard, W.A. Multiplicity of DNA Single-Strand Breaks Produced in pUC18 Exposed to the Direct Effects of Ionizing Radiation. *Radiat. Res.* **2008**, *170*, 156-162.
27. Purkayastha, S.; Milligan, J.R.; Bernhard, W.A. Correlation of Free Radical Yields with Strand Break Yields Produced in Plasmid DNA by the Direct Effect of Ionizing Radiation. *J. Phys. Chem. B* **2005**, *109*, 16967-16973.
28. Yu, H.; Kondo, Y.; Fujii, K.; Yokoya, A.; Yamashita, S. Establishment of a Method for Investigating Direct and Indirect Actions of Ionizing Radiation Using Scavenger-Free Plasmid DNA. *Radiat. Res.* **2022**, *197*, 594-604.
29. Shiraishi, I.; Shikazono, N.; Suzuki, M.; Fujii, K.; Yokoya, A. Efficiency of Radiation-Induced Base Lesion Excision and the Order of Enzymatic Treatment. *Int. J. Radiat. Biol.* **2017**, *93*, 295-302.

30. Vyšín, L.; Pachnerová Brabcová, K.; Štěpán, V.; Moretto-Capelle, P.; Bugler, B.; Legube, G.; Cafarelli, P.; Casta, R.; Champeaux, J.P.; Sence, M.; et al. Proton-Induced Direct and Indirect Damage of Plasmid DNA. *Radiat. Environ. Biophys.* **2015**, *54*, 343-352.
31. Yokoya, A.; Cunniffe, S.M.T.; O'Neill, P. Effect of Hydration on the Induction of Strand Breaks and Base Lesions in Plasmid DNA Films by γ-Radiation. *J. Am. Chem. Soc.* **2002**, *124*, 8859-8866.
32. Yokoya, A.; Cunniffe, S.M.T.; Stevens, D.L.; O'Neill, P. Effects of Hydration on the Induction of Strand Breaks, Base Lesions, and Clustered Damage in DNA Films by α-Radiation. *J. Phys. Chem. B* **2003**, *107*, 832-837.
33. Urushibara, A.; Shikazono, N.; O'Neill, P.; Fujii, K.; Wada, S.; Yokoya, A. LET Dependence of the Yield of Single-, Double-Strand Breaks and Base Lesions in Fully Hydrated Plasmid DNA Films by $^{4}He^{2+}$ Ion Irradiation. *Int. J. Radiat. Biol.* **2008**, *84*, 23-33.
34. Ushigome, T.; Shikazono, N.; Fujii, K.; Watanabe, R.; Suzuki, M.; Tsuruoka, C.; Tauchi, H.; Yokoya, A. Yield of Single- and Double-Strand Breaks and Nucleobase Lesions in Fully Hydrated Plasmid DNA Films Irradiated with High-LET Charged Particles. *Radiat. Res.* **2012**, *177*, 614-627.
